# Reducing Capacity, Chlorogenic Acid Content and Biological Activity in a Collection of Scarlet (*Solanum aethiopicum*) and Gboma (*S. macrocarpon*) Eggplants

**DOI:** 10.3390/ijms151017221

**Published:** 2014-09-26

**Authors:** Mariola Plazas, Jaime Prohens, Amparo Noelia Cuñat, Santiago Vilanova, Pietro Gramazio, Francisco Javier Herraiz, Isabel Andújar

**Affiliations:** Instituto de Conservación y Mejora de la Agrodiversidad Valenciana, Universitat Politècnica de València, Camino de Vera 14, 46022 Valencia, Spain; E-Mails: maplaav@btc.upv.es (M.P.); amcuata@etsiamn.upv.es (A.N.C.); sanvina@upvnet.upv.es (S.V.); pietrogramazio@yahoo.it (P.G.); fraherga@upvnet.upv.es (F.J.H.); isanpe@upvnet.upv.es (I.A.)

**Keywords:** *Solanum aethiopicum*, *Solanum macrocarpon*, phenolic acids, chlorogenic acid, cultivar groups, diversity, nitric oxide, bioactive properties, breeding

## Abstract

Scarlet (*Solanum aethiopicum*) and gboma (*S. macrocarpon*) eggplants are important vegetables in Sub-Saharan Africa. Few studies have been made on these crops regarding the diversity of phenolic content and their biological activity. We have studied the reducing activity, the chlorogenic acid and other phenolic acid contents in a collection of 56 accessions of scarlet eggplant, including the four cultivated groups (Aculeatum, Gilo, Kumba, Shum) and the weedy intermediate *S. aethiopicum*-*S. anguivi* types, as well as in eight accessions of gboma eggplant, including the cultivated *S. macrocarpon* and its wild ancestor, *S. dasyphyllum*. A sample of the accessions evaluated in this collection has been tested for inhibition of nitric oxide (NO) using macrophage cell cultures. The results show that there is a great diversity in both crops for reducing activity, chlorogenic acid content and chlorogenic acid peak area (% of total phenolic acids). Heritability (H^2^) for these traits was intermediate to high in both crops. In all samples, chlorogenic acid was the major phenolic acid and accounted for more than 50% of the chromatogram peak area. Considerable differences were found among and within groups for these traits, but the greatest values for total phenolics and chlorogenic acid content were found in *S. dasyphyllum*. In most groups, reducing activity was positively correlated (with values of up to 0.904 in the Aculeatum group) with chlorogenic acid content. Inhibition of NO was greatest in samples having a high chlorogenic acid content. The results show that both crops are a relevant source of chlorogenic acid and other phenolic acids. The high diversity found also indicates that there are good prospects for breeding new scarlet and gboma eggplant cultivars with improved content in phenolics and bioactive properties.

## 1. Introduction

Selection of vegetables with improved content in bioactive phenolics is a breeding objective of an increasing number of genetic improvement programs aimed at developing new varieties with enhanced functional properties [1,2]. Dietary phenolics of vegetables and of other plant products have been shown to have bioactive properties beneficial for human health, resulting from, among others, free-radical scavenging properties, regulation of enzymatic activity or modulation of several cell signaling pathways [3,4,5,6,7].

Among phenolic compounds, phenolic acids exert a potent antioxidant activity through the interaction with reactive oxygen and nitrogen species by several mechanisms [8]. This strong reducing capacity is the key to their biological activity [9]. Among these phenolic acids, chlorogenic acid (5-*O*-caffeoyl-quinic acid) is abundant in vegetables [2,10,11] and has been shown to present multiple beneficial properties, including analgesic, anti-carcinogenic, anti-diabetic, anti-inflammatory, anti-microbial, anti-obesity, cardioprotective, hypotensive and neuroprotective effects [2,6,12,13,14,15,16,17,18]. Furthermore, chlorogenic acid is highly bioavailable for humans [18]. Therefore, selecting vegetable varieties with higher chlorogenic acid content may be of interest [2].

Common eggplant (*Solanum melongena* L.) has been reported as having a very high content of chlorogenic acid, which constitutes the major phenolic compound in the fruit flesh [2,14,19,20,21,22], and some breeding programs for improving total phenolics and chlorogenic acid content have been started [2,21,22]. Apart from the common eggplant, there are two cultivated eggplants native to Africa, namely the scarlet eggplant (*S. aethiopicum* L.) and the gboma eggplant (*S. macrocarpon* L.), which, despite their importance in Sub-Saharan Africa [23], have largely remained neglected. Both crops are hypervariable at the morphological level, in particular in the case of the scarlet eggplant [23,24,25,26,27,28,29,30]. Four cultivar groups of *S. aethiopicum* are recognized: Aculeatum (used as ornamental), Gilo (used for the fruits), Kumba (used for the fruits and leaves) and Shum (used for the leaves) [24,25,30]. Furthermore, weedy plants of semi-domesticated forms that are intermediate in characteristics between the cultivated *S. aethiopicum* and its wild ancestor, *S. anguivi* L., are commonly harvested [25,30,31]. For the purposes of this paper, these intermediate forms are referred to as *aethiopicum-anguivi*. The gboma eggplant, *S. macrocarpon*, which is cultivated for its fruits and leaves, is generally not differentiated into cultivar groups, although the wild ancestor, *S. dasyphyllum* Schum and Thon., is included in the gboma eggplant complex [26]. The wild *S. dasyphyllum* is clearly distinguished from the cultivated *S. macrocarpon* for having greater prickliness and smaller fruits and is mostly used as medicinal [23,26].

Few efforts have been devoted to evaluating the phenolic content of scarlet and gboma eggplants [19,22,29]. These studies have shown that, like common eggplant, both species present high levels of total phenolics and of chlorogenic acid. However, the diversity of these crops for their reducing activity, phenolic acid content or their relationship with biological activity has been barely studied. The largest study on scarlet eggplant diversity related to this subject was performed by Sunseri *et al.* [29], who evaluated 70 accessions of scarlet eggplant for chlorogenic acid content and found a wide range of variation, from 0.20 to 9.88 g/kg. However, the results were part of a general study of characterization and did not involve studying differences among groups or other related traits, like reducing activity or biological activity of varieties having different chlorogenic acid concentrations [29]. Furthermore, Stommel and Whitaker [19] evaluated 13 accessions of *S. aethiopicum* for phenolic acids in a general study of the diversity of phenolic acid composition in common eggplant and found a range of variation for chlorogenic acid from 1.09 to 3.52 g/kg. For the gboma eggplant, we know no studies for the diversity of reducing activity, chlorogenic acid content or biological activity. As a consequence, it is desirable to undertake a detailed investigation on the functional properties and compounds and biological activity of both the scarlet and gboma eggplants. Apart from providing information relevant on the properties of both crops, this knowledge will be of interest for selection and breeding of varieties of both crops with improved functional properties. In addition, the common, scarlet and gboma eggplants can be intercrossed, giving hybrids of intermediate fertility [32,33,34,35]. Therefore, the three cultivated eggplant species might be used as genetic resources for reciprocal breeding [35,36], including introgression of functional quality traits [37].

In this work, we characterize the total reducing activity, as well as the chlorogenic acid and other phenolic acid content in a collection of scarlet and gboma eggplants from different groups. Moreover, in a selected set of accessions, we carried out a study of the biological activity *in vitro* in macrophages. The objective is to provide relevant information on the reducing activity, chlorogenic acid content and their relationship and to test the biological activity of the extracts of scarlet and gboma eggplants. This information will be useful for developing eggplants with improved functional properties, *i.e.*, with higher content in chlorogenic acid and enhanced antioxidant and biological activity.

## 2. Results and Discussion

### 2.1. Variation among Accessions

The mean values for the total reducing capacity of scarlet eggplant and gboma eggplant collections were 7.45 and 11.16 g/kg of chlorogenic acid equivalents (*i.e*., the concentration of pure chlorogenic acid concentration that would be required for accounting for this reducing activity), respectively (Table 1). For chlorogenic acid content, the average values were, respectively, 1.51 and 1.66 g/kg. These values for total reducing capacity and chlorogenic acid content reveal that, as with the common eggplant [2,19,21,38], both the scarlet and gboma eggplants have high levels of total reducing activity and chlorogenic acid content, although there is not a perfect correlation between these two traits.

**Table 1:** Percentage of the total sum of squares for the effects of accession and residual, global mean, minimum and maximum accession means, average standard error for accession means (SE), coefficient of phenotypic variation (CV_P_), coefficient of genotypic variation (CV_G_) and heritability (H^2^) for total reducing capacity (expressed as equivalents of chlorogenic acid, CGA), chlorogenic acid content and percentage of peak area (for high-performance liquid chromatography at 325 nm) corresponding to chlorogenic acid for the fruit traits evaluated in a collection of 56 accessions of scarlet eggplant and eight accessions of gboma eggplant.

Trait	Sum of Squares (%)		Mean	Minimum	Maximum	SE	CV_P_ (%)	CV_G_ (%)	H^2^
	Accession	Residual							
Scarlet eggplant (*n* = 56)									
Total reducing capacity (equivalents of CGA; g·kg^−1^)	87.55 ***	12.45	7.45	3.83	16.92	0.62	28.61	21.79	0.58
Chlorogenic acid (g·kg^−1^)	82.76 ***	17.24	1.51	0.21	4.47	0.19	39.92	27.69	0.48
Chlorogenic acid peak area (%)	41.56 ***	58.44	78.62	50.3	95.3	2.82	10.06	6.07	0.36
Gboma eggplant (*n* = 8)									
Total reducing capacity (equivalents of CGA; g·kg^−1^)	84.34 ***	15.66	11.16	7.15	22.69	1.09	32.37	23.82	0.54
Chlorogenic acid (g·kg^−1^)	94.89 ***	5.11	1.66	0.48	4.87	0.15	46.26	41.58	0.81
Chlorogenic acid peak area (%)	73.65 ***	26.35	60.87	50.4	71.5	2.03	9.41	5.73	0.37

*** Indicates significant at *p* < 0.001.

Our values for chlorogenic acid are similar to those obtained by Stommel and Whitaker [19]. However, Sunseri *et al.* [29] found an average value for chlorogenic acid in scarlet eggplant of around two-fold higher than our values. Differences in extraction procedures and environmental effects, which are important for phenolic content in eggplant, as revealed in a recent study [39], as well the stage of fruit harvesting might account for these differences [39,40,41,42,43]. Wide ranges of variation were found for both reducing activity and chlorogenic acid content in the two collections, with differences of up to 4.4- and 3.2-fold in scarlet and gboma eggplants, respectively, for total reducing capacity and of up to 21.3- and 10.1-fold in scarlet eggplant and gboma eggplants, respectively, for chlorogenic acid content (Table 1). This is in agreement with the results obtained by Sunseri *et al.* [29], who found a range of variation of 49.6-fold for chlorogenic acid content in a collection of 70 accessions of scarlet eggplant. The percentage of the sums of squares of accession for both traits and in both crops was always above 80%, with differences among accessions being highly significant (*p* < 0.001) (Table 1). A large range of variation for both reducing activity measured with the Folin–Ciocalteu reagent and for chlorogenic acid content has also been found in collections of common eggplant [19,21,37,40,44], suggesting that, in general, reducing activity and chlorogenic acid content levels are very variable in eggplants and, therefore, amenable to selection [45].

Average values of the chlorogenic acid peak area percentage in the HPLC chromatograms were of 78.62% for scarlet eggplant and of 60.87% in gboma eggplant (Table 1). In all accessions, the chlorogenic acid peak area accounted for more than 50% of the total peak area in the chromatogram, with maximum values of 93.3% in scarlet eggplant (in one accession of the Gilo group) and 71.5% in gboma eggplant. This is in agreement with the results of Stommel *et al.* (2003), who found that between 63.4% and 96.0% of the total phenolic acid content of 13 accessions *S. aethiopicum* and 73.0% of the total phenolic content of a single accession of *S. macrocarpon* as evaluated by HPLC corresponded to chlorogenic acid. Values obtained are also similar to those of *S. melongena* [19,22,42]. Differences among accessions in both collections were highly significant (*p* < 0.001), although the percentage of sums of squares of accession was lower than for total reducing activity and chlorogenic acid content (Table 1). Furthermore, the coefficients of phenotypic and genotypic variation for the chlorogenic acid peak area were much lower than those of total reducing activity and chlorogenic acid (Table 1).

Broad-sense heritability (H^2^) values between 0.3 and 0.7 are considered as moderate; while those above 0.7 are regarded as high. In both the scarlet and gboma eggplant collections, H^2^ values were moderate for the three traits evaluated, except for chlorogenic acid in the gboma eggplant, in which they were high (0.81) (Table 1). Prohens *et al.* [21] obtained H^2^ values of 0.50 for reducing activity measured with the Folin–Ciocalteu reagent in a collection of common eggplant, which are similar to the values obtained by us in scarlet and gboma eggplants. The H^2^ values obtained for scarlet and gboma eggplant for the three traits indicate that selection will be efficient, and therefore, there are good prospects for a significant genetic advance in selection and breeding programs [45]. This is an indication that selection for total reducing activity or chlorogenic acid content may be more efficient than selection for modifying the phenolics acid profile. However, given the positive correlation between total phenolics and chlorogenic acid content (see Section 2.3), selection for one of these traits will also result in the indirect selection for the other [45].

### 2.2. Differences between Groups

Of the five scarlet and two gboma eggplants groups, the highest total reducing capacity was found in the wild gboma eggplant, *S. dasyphyllum*, which, with a value of 22.69 g/kg of chlorogenic acid equivalents, had significantly (*p* < 0.05) higher levels than the rest of groups studied (Table 2). The scarlet eggplant Kumba group ranked second, with values (12.86 g/kg) significantly higher than those of the rest of scarlet eggplant groups. When considering chlorogenic acid content, again, *S. dasyphyllum* presented the highest value (4.87 g/kg), being significantly higher than that of the rest of groups (Table 2). *Solanum dasyphyllum* was followed by the scarlet eggplant group, Shum (3.03 g/kg), which had values significantly higher than those of the scarlet eggplant groups, Aculeatum, Gilo and Kumba, and of the gboma eggplant, *S. macrocarpon* (Table 2). In particular, *S. dasyphyllum* presents values much higher than the rest of groups for both traits, indicating that this species is a source of variation of considerable interest for improving the content of the cultivated *S. macrocarpon*. However, before using *S. dasyphyllum* in breeding programs, it would be advisable to study the presence in this wild species and subsequent generations of potentially toxic compounds, like glycoalkaloids. Furthermore, the availability of a broad range of variation may be useful for developing populations using parents with contrasting values for the study of the inheritance, mapping Quantitative Trait Loci (QTL) and validating candidate genes for reducing capacity and chlorogenic acid content [46].

**Table 2:** Means and range for total reducing capacity (expressed as equivalents of chlorogenic acid), chlorogenic acid content (CGA) and percentage of peak area (for high-performance liquid chromatography at 325 nm) corresponding to chlorogenic acid.

Group	*n*	Total Reducing Capacity (Equivalents of CGA; g·kg^−1^) ^z^		Chlorogenic Acid (g·kg^−1^) ^z^		Chlorogenic Acid Peak Area (%) ^z^	
		Mean	Range	Mean	Range	Mean	Range
Scarlet eggplant (*n* = 56)							
Aculeatum	5	7.39 c	6.02–8.64	1.28 c	0.70–2.16	79.4 a	76.5–82.3
*aethiopicum-anguivi*	6	8.01 c	6.23–11.55	2.25 bc	1.17–4.47	78.7 a	67.6–87.0
Gilo	34	6.02 c	3.83–16.45	1.46 c	0.21–3.69	79.6 a	50.3–93.3
Kumba	9	12.86 b	9.41–16.92	0.99 c	0.23–1.55	72.5 ab	58.8–82.9
Shum	2	5.87 c	3.86–7.87	3.03 b	2.21–3.83	87.6 a	86.0–89.2
Gboma eggplant (*n* = 8)							
*S. dasyphyllum*	1	22.69 a	–	4.87 a	–	50.4 c	–
*S. macrocarpon*	7	9.51 bc	7.15–16.03	1.20 c	0.48–1.98	62.4 bc	54.3–71.5

^z^ Varietal group means within columns separated by different letters are significantly different according to the Duncan multiple range test at *p* ≤ 0.05.

**Figure 1:**
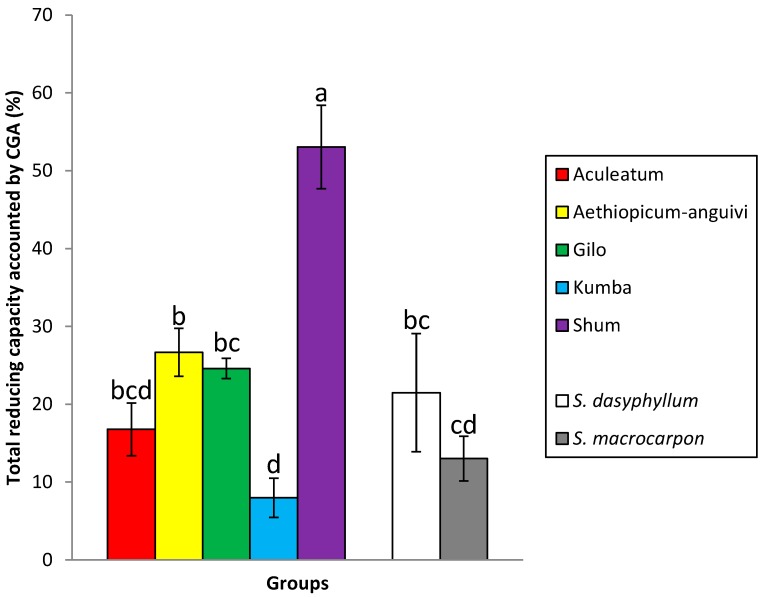
Percentage of total reducing capacity explained by CGA in the different scarlet eggplant (Aculeatum, *aethiopicum-anguivi*, Gilo, Kumba and Shum) and gboma eggplant (*S. dasyphyllum* and *S. macrocarpon*) groups. Bars represent ±standard error of the mean for each of the groups obtained from an ANOVA. Different letters indicate significantly different means at *p* ≤ 0.05 according to Duncan’s multiple range test.

Unexpectedly, the Kumba group, which displayed the highest average value among scarlet eggplant groups for the total reducing capacity, had low values for chlorogenic acid content (Table 2). Conversely, the Shum group, which had low average values for total reducing capacity, presented high chlorogenic acid content. This results in differences among groups in the percentage of total reducing capacity accounted for by chlorogenic acid (Figure 1). In this respect, while in the Kumba group, chlorogenic acid accounts for less than 10% of the total reducing capacity, in the case of the group, Shum, it accounts for more than 50%. The rest of groups present values between 13.0% (*S. macrocarpon*) and 26.6% (*aethiopicum-anguivi*) (Figure 1). In common eggplant, it has been found that chlorogenic acid measured by HPLC generally accounts for between 15% and 75% of the reducing activity of common eggplant measured with the Folin–Ciocalteu reagent [42,47,48]. Similarly to our results, in common eggplant, considerable differences among accessions and between groups of accessions in the percentage of reducing activity accounted for by chlorogenic acid have been found [42,47].

**Figure 2:**
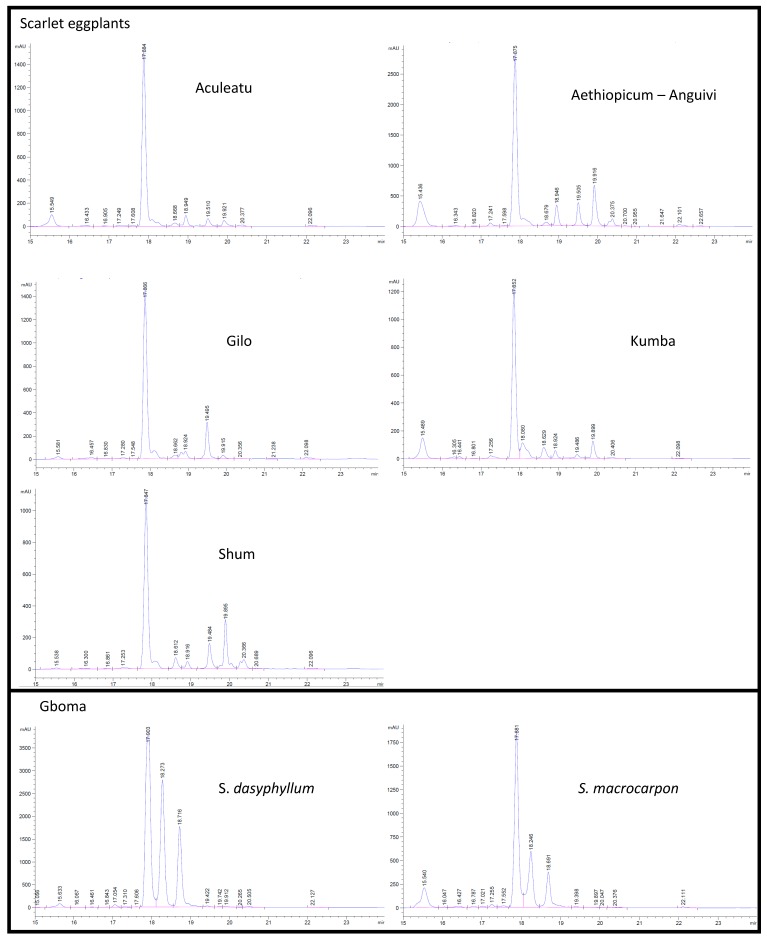
Representative C18-high performance liquid chromatography chromatograms of phenolic compounds (detected at 325 nm) in methanolic extracts of accessions of scarlet eggplant and gboma eggplant groups. The chlorogenic acid (CGA) peak is indicated. Note that different groups may have different peak scales.

The percentage of peak area corresponding to chlorogenic acid in the HPLC chromatogram also presented significant differences among groups (Table 2). In particular, the gboma eggplant groups had values significantly lower than those of the scarlet eggplant groups (with the exception of a non-significant difference between *S. macrocarpon* and the Kumba group). While in all groups, chlorogenic acid was the predominant phenolic acid, the HPLC profiles presented considerable differences, in particular between the scarlet eggplant groups and the gboma eggplant groups (Figure 2). This confirms that differences among eggplant relatives for the phenolic compound profile are important and may have a potential use in interspecific chemotaxonomy [49].

The within group range of variation for total reducing capacity, chlorogenic acid content and chlorogenic acid peak area was large, in particular for the Gilo group, which was the group with a larger number of accessions studied (Table 2). The Kumba group, despite being represented by nine accessions, did not contain any accessions with high chlorogenic acid levels, with eight of the nine accessions in this group having values below the average value for the whole scarlet eggplant collection (1.51 g/kg). The availability of a wide range of variation within each of the groups has important implications for breeding, as it shows that it may be possible to select within each of the groups accessions with improved levels of the desired trait/s [45].

### 2.3. Relationship between Total Reducing Capacity and Chlorogenic Acid Content

**Figure 3:**
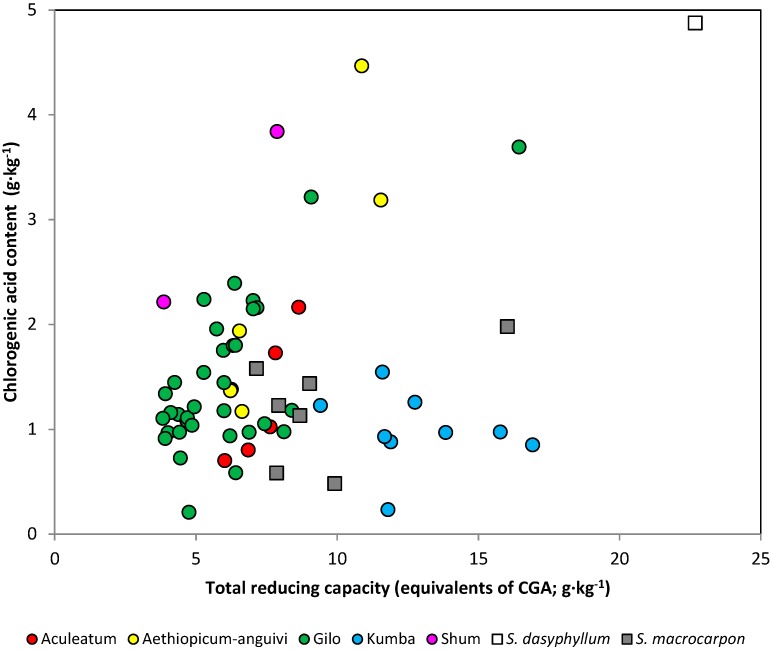
Relationship between the total reducing capacity (expressed as equivalents of CGA; *x*-axis) and CGA content (*y*-axis) for the individual accessions of the different scarlet eggplant and gboma eggplant groups.

When considering the whole collection, the linear correlation coefficient between accession means for the total reducing capacity and chlorogenic acid content in all the accessions was significant, but presented a relatively low value (*r* = 0.370) (Table 3). Consequently, the coefficient of determination (*r*^2^) was also low, revealing that only 13.7% of the variation in total reducing activity was explained by the variation in chlorogenic acid. This is contrast with previous results in common eggplant, in which higher values for this correlation have been found [47]. However, a closer examination of the data revealed that this low value might be caused by an admixture of different groups in the analysis, each of which has a different relationship pattern (Figure 3). This may result in low correlation values when all accessions are considered together [50]. In this respect, the Kumba group presented a total reducing capacity-chlorogenic acid content relationship different from that of the other groups (Figure 1), which resulted in a non-significant correlation coefficient between total reducing capacity and chlorogenic acid content when the whole collection of scarlet eggplant accessions is considered (Table 3). However, when the correlation analysis was performed separately for each group, we found that the Aculeatum, *aethiopicum-anguivi* and Gilo groups presented high within-group correlation values, above 0.9 for the two former and of 0.675 for the latter (Table 3). These values are in agreement with previous results in common eggplant in which in a collection of 18 accessions, the correlation between reducing activity measured with the Folin–Ciocalteu reagent and CGA was moderate at 0.633 [47].

**Table 3:** Coefficients (coef.) of correlation (*r*) and determination (*r*^2^; %), *F*-ratio and significance (probability (prob.) of *F*) for the linear model for the relationship between total reducing activity and chlorogenic acid for the 64 accessions of scarlet eggplant and gboma eggplant studied.

Group	*n*	Coef. Correlation	Coef. Determination (%)	*F*-Ratio	Prob. *F*
All accessions	64	0.370	13.7	9.83	0.0026
Scarlet eggplant	56	0.197	3.9	2.18	0.1453
Aculeatum	5	0.904	81.6	13.34	0.0354
*aethiopicum-anguivi*	6	0.901	81.1	17.17	0.0143
Gilo	34	0.675	45.5	26.72	<0.0001
Kumba	9	−0.179	3.2	0.23	0.6451
Shum ^a^	2	–	–	–	–
Gboma eggplant	8	0.893	79.7	23.59	0.0028
*S. dasyphyllum* ^a^	1	–	–	–	–
*S. macrocarpon*	7	0.499	24.9	1.65	0.2548

^a^ For these groups, no degrees of freedom were available to evaluate the significance of the linear correlation.

The high values obtained for the total reducing activity and chlorogenic acid in the Aculeatum, *aethiopicum-anguivi* and Gilo groups have implications for breeding, as they indicate that in these groups, selection for one of these two traits will result in indirect selection for the other [46]. However, for the Kumba group, the correlation value obtained was non-significant, revealing that in this group, variation in chlorogenic acid content did not contribute to explaining the variation in total reducing capacity. As a consequence, compounds other than chlorogenic acid must play a major role in the antioxidant capacity of the Kumba group. When considering that the *S. macrocarpon* accessions were studied separately, the correlation value was of 0.499, which was somewhat lower than that of the regular groups of scarlet eggplant (Table 3).

Apart from chlorogenic acid, other phenolic acids present in the fruit different from chlorogenic acid and that were detected in the HPLC chromatogram must also have had a contribution to the reducing activity [19,43]. Phenolic acids have different reducing activities [3], and some compounds present at low concentrations might have a relevant role in accounting for the total reducing capacity. For example, hydroxybenzoic acid has a Trolox equivalent antioxidant capacity (TEAC) more than 30-times lower than that of rosmarinic acid [3]. Furthermore, other non-phenolic antioxidants, proteins and inorganic ions present in the eggplant fruit flesh may react with the Folin–Ciocalteu reagent [51,52,53], which may contribute to the total reducing activity. However, further studies should be made to identify precisely the different compounds that play a major role in accounting for the total reducing activity apart from chlorogenic acid.

### 2.4. Biological Activity

Both chronic inflammatory diseases and cardiovascular diseases are associated with an altered nitric oxide (NO) production, a free radical involved in many physiological processes in the human body [54]. Dietary polyphenols have shown to exhibit beneficial biological activities, such as free-radical scavenging, regulation of enzymatic activity and modulation of several cell signaling pathways, which explain their proven antioxidant, anti-inflammatory, anticarcinogenic and preventive effects on coronary diseases [6,54].

**Figure 4:**
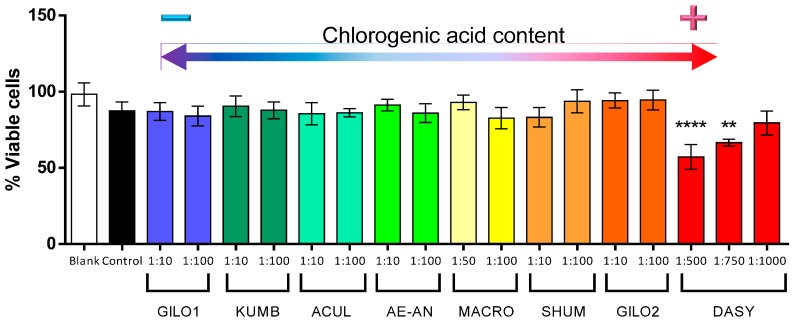
Percentage of viable cells of RAW 264.7 macrophages incubated in different dilutions of methanolic extracts of scarlet and gboma eggplant accessions (see Table 5 for code descriptions and the chlorogenic acid content of individual accessions). Accessions have been ordered according to the chlorogenic acid content of the pure extracts, with the lowest values to the left and the highest values to the right. Bars represent ±standard error of the mean. Columns tagged with asterisks indicate that the mean values are significantly different from the control (** *p* < 0.01; **** *p* < 0.0001) according to Dunnett’s multiple comparison test.

In order to test the effect of the eggplant extracts in nitric oxide production, we first evaluated the toxicity of the different extracts of several accessions to determine the non-toxic dilutions. As shown in Figure 3, ten-fold dilutions or higher showed no toxicity, except for the extracts of the gboma eggplant accessions, which had to be used at a 1:50 dilution or higher in the case of *S. macrocarpon*, and 1:1000 in the case of *S. dasyphyllum* (Figure 4). This may suggest that in the gboma eggplants, compounds other than phenolic acids may account for this toxicity. In this respect, Sánchez-Mata *et al.* [55] found that gboma eggplants had higher glycoalkaloid content than scarlet eggplants. However, further studies should be conducted to identify the underlying cause or compounds reducing cell viability in the gboma eggplants.

**Figure 5:**
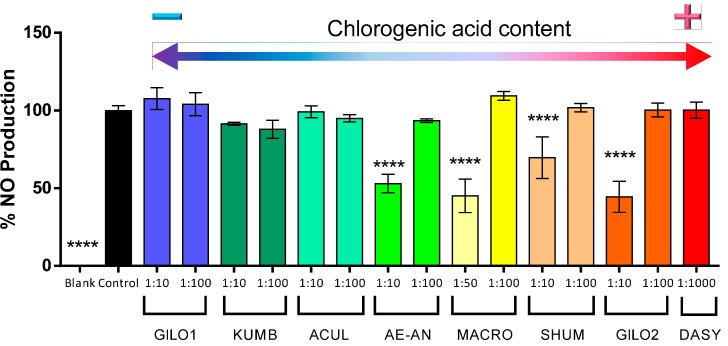
Percentage of NO production of RAW 264.7 macrophages incubated in different non-cytotoxic dilutions of methanolic extracts of scarlet and gboma eggplant accessions (see Table 5 for code descriptions and the chlorogenic acid content of individual accessions). Accessions have been ordered according to the chlorogenic acid content of the pure extracts, with the lowest values to the left and the highest values to the right. Bars represent ±standard error of the mean. Columns tagged with asterisks indicate that the mean values are significantly different from the control (**** *p* < 0.0001) according to Dunnett’s multiple comparison test.

Hwang *et al.* [56] recently demonstrated *in vitro* in RAW 264.7 macrophages that chlorogenic acid significantly inhibits NO production by inhibiting the inducible nitric oxide synthase without any cytotoxicity. As shown in Figure 4, our results demonstrate that those accessions with a higher content in chlorogenic acid are able to significantly reduce about 50% of the LPS-induced NO production, and this NO inhibition occurs in a dose-dependent manner. This is true in all cases, except in the case of *S. dasyphyllum*. The *S. dasyphyllum* accession shows the highest total reducing activity and the highest content in chlorogenic acid (Table 2), but a very high dilution (1:1000) had to be tested, given its cytotoxicity (Figure 5); and this may be the cause of the lack of inhibition of NO production.

The results obtained reveal that there are accessions that present high reducing activity, chlorogenic acid content and high values for NO production inhibition, in particular some accessions of the Gilo and Shum groups of *S. aethiopicum*, as well as in *S. macrocarpon*. These materials could be selected for their direct use or for being incorporated in breeding programs aimed at developing eggplants with healthier properties.

## 3. Experimental Section

### 3.1. Plant Material

Fifty-six accessions of scarlet eggplant and eight accessions of gboma eggplant were used for the present study (Table 4). Five plants per accession were grown in Valencia (Spain) in the open field during the summer season of 2013 using the standard horticultural practices used for common eggplant. These accessions had been previously characterized morphologically by Plazas *et al.* [30] and assigned to their respective groups: Aculeatum (5), *aethiopicum-anguivi* (6), Gilo (34), Kumba (9) and Shum (2) for the scarlet eggplant; and *S. dasyphyllum* (1) and *S. macrocarpon* (7) for the gboma eggplant.

**Table 4:** Scarlet eggplant and gboma eggplant groups evaluated, number of accessions and typical characteristics of the fruit of each of the groups [23,24,25,26,30].

Group	*n*	Type	Common Use	Fruit Weight (g)	Fruit Shape	Fruit Diameter (cm)	Fruit Grooves	Fruit Locules	Calyx Prickliness
Scarlet eggplant (*n* = 56)									
Aculeatum	5	Cultivated	Ornamental	20–40	Flattened	3–8	Many	4–10	Very high
*aethiopicum-anguivi*	6	Weedy ^a^	Medicinal	3–8	Ellipsoid	1–3	None to few	2–3	Absent to low
Gilo	34	Cultivated	Food (fruits)	10–70	Subspherical to ellipsoid	3–8	None to few	2–6	Absent to low
Kumba	9	Cultivated	Food (fruits and leaves)	50–350	Flattened	5–12	Very many	10–20	None
Shum	2	Cultivated	Food (leaves)	2–6	Round	2–3	None to few	2–4	None
Gboma eggplant (*n* = 8)									
*S. dasyphyllum*	1	Wild	Medicinal	15–30	Subspherical	3–5	None	2–5	Very high
*S. macrocarpon*	7	Cultivated	Food (fruits and leaves)	50–150	Subspherical	5–9	None	4–6	Absent to low

^a^ Found as a non-cultivated plant in disturbed environments.

One sample of fruit, consisting of either a minimum of 250 g or five fruits (for the small fruited accessions), was obtained for each plant. Commercially ripe fruits (*i.e*., physiologically immature at the breaker stage) were used [30]. Fruits were brought to the laboratory, washed, and a slice of a 1–2 cm-wide longitudinal section from stem to blossom end was cut from the middle of the fruit. The excised tissue was frozen in liquid N_2_ and lyophilized. The lyophilized tissue of the fruit from an individual plant was powdered and pooled as a single sample. The powdered tissue of each sample was used for the analyses.

### 3.2. Chemical Analysis

#### 3.2.1. Total Reducing Capacity

Total reducing capacity was determined according to the Folin–Ciocalteu procedure [57,58]. For each sample, 0.125 g of the lyophilized tissue was extracted with 15 mL of acetone:water:glacial acetic acid (70:29.5:0.5, *v*/*v*/*v*) for 24 h under continuous stirring at room temperature. The extracted sample was then centrifuged at 3500 rpm for 5 min in an Eppendorf 5804 R centrifuge (Eppendorf, Hamburg, Germany), and 1.5 mL of the supernatant were pipetted, poured on Eppendorf tubes and stored at −20 °C until analyzed. Thawed samples were centrifuged at 10,000 rpm for 5 min, and 65 μL of the supernatant were mixed with 0.5 mL diluted (10%, *v*/*v*) Folin–Ciocalteu reagent (Sigma-Aldrich Chemie, Steinheim, Germany) and allowed to stand at room temperature for 5 min. Subsequently, 0.5 mL of sodium carbonate (60 g/L) were added to the mixture. After 90 min at room temperature, absorbance was measured at 750 nm in an iMark microplate spectrophotometer (Bio-Rad, Herts, UK). Chlorogenic acid (Sigma-Aldrich Chemie) was used as a standard, and total reducing capacity was expressed as chlorogenic acid equivalents in g/kg of dry weight (Table 1 and Table 2).

#### 3.2.2. Chlorogenic Acid and Other Free Phenolic Acids

Chlorogenic acid and other phenolic acids (hydroxycinnamic acid conjugates) were extracted according to Helmja *et al.* [59]. Lyophilized samples (0.1 g) were homogenized in 1.8 mL of methanol:water (80:20, *v*/*v*) plus 0.1% (*w*/*v*) of 2,3-*tert*-butyl-4-hydroxyanisole (BHT). The total extract was vortexed vigorously, sonicated for 1 h at room temperature and then centrifuged at 2000 rpm for 3 min in an Eppendorf 5804 R centrifuge. The supernatant was filtered through 0.2-µm polytetrafluoroethylene (PTFE) membrane filters. Standard solutions of chlorogenic acid were prepared using the same protocol.

Determination of the content of chlorogenic acid and other hydroxycinnamic acid derivatives was performed by high-performance liquid chromatography (HPLC) according to the protocol of Luthria and Mukhopadhyay [41]. Extracts were analyzed on a HPLC 1220 Infinity LC System (Agilent Technologies, Santa Clara, CA, USA) operated by the OpenLAB CDS ChemStation Edition software package (Agilent Technologies). Aliquots of 10 μL were injected with the 1220 Infinity LC System automatic sampler into a ZORBAX Eclipse Plus C18 (3.5 μm; 4.6 mm × 12.5 mm; Agilent Technologies) column protected by a ZORBAX Eclipse Plus C18 guard column (5 μm; 4.6 mm × 12.5 mm; Agilent Technologies). The method used was a modification of that described by Prohens *et al.* (2013). The binary gradient consisted of 0.1% formic acid (Solvent A) and methanol (Solvent B). The mobile phase gradient was as follows: 0 min, 95 A:5 B at 0.5 mL/min; 0–5 min linear increase to 10% B at 0.5 mL/min; 5–10 min, linear increase to 20% B at 0.5 mL/min; 10–18 min, linear increases to 83% B and 0.5 mL/min; 18–23 min, linear increase to 100% B at 0.5 mL/min; 23–27 min, 100% B at 1.0 mL/min; 27–30 min, decrease to 5% B at 1.0 mL/min; 30-40 min, 95 A:5 B at 0.5 mL/min. Quantification was based on absorbance at 325 nm. The concentration of chlorogenic acid in the extracted samples was calculated using the developed calibration curves. The calibration curve was calculated using unweighted linear regression analysis, and fit to linearity was evaluated with the *r*^2^ value (*r*^2^ > 0.99). The chlorogenic acid peak area and the total peak area of other phenolic acids (hydroxycinnamic acid conjugates) were determined and used to calculate the percentage of total peak area corresponding to chlorogenic acid (Table 1 and Table 2).

### 3.3. Biological Assays

#### 3.3.1. Cell Cultures

The murine macrophage cell line RAW 264.7 (ECACC, Salisbury, UK) was used for all *in vitro* experiments. The cells were maintained in Dulbecco’s modified Eagle’s medium (Life Technologies, Carlsbad, CA, USA) supplemented with 10% fetal bovine serum (Life Technologies), penicillin (100 U/mL, Life Technologies) and streptomycin sulfate (100 mg/mL, Life Technologies) in a humidified 5% CO_2_ atmosphere.

#### 3.3.2. Preparation of Extracts for Biological Assays

A set of eight accessions from the different groups (one for each group and two for the large Gilo group) was chosen based in their differences in chlorogenic acid content (Table 5). Lyophilized fruit samples (1 g) were homogenized in 30 mL of methanol and extracted in an ultrasonic bath (Elmasonic S30, Elma, Singen, Germany) for 1 h. Extracted samples were centrifuged at 2000 rpm for 3 min in an Eppendorf 5804 R centrifuge, and the supernatant was collected and filtered with PTFE filters. Samples were dried in a vacuum centrifuge (SpeedVac^®^, Thermo Scientific, Waltham, MA, USA) and redissolved in ultrapure water (MilliQ Millipore, Molsheim, France). Finally, they were filtered through 0.2-µm sterile PTFE filters, and 1:10, 1:50 and 1:100 dilutions in sterile phosphate buffered saline were prepared.

#### 3.3.3. Cell Viability Assay

The effect of each extract on cell viability was evaluated with the 3-[4,5-dimethylthiazol-2-yl]-2,5-diphenyl-tetrazolium bromide (MTT) assay. In brief, murine RAW 264.7 macrophages were exposed to different dilutions of each extract in a 96-well microplate for 24 h, after which 20 µL per well of a 5 mg/mL solution of MTT (Sigma-Aldrich Chemie) was added, and cells were incubated for 40 min at 37 °C until blue deposits of formazan were visible. Metabolically active cells are able to transform MTT into formazan; therefore, the greater the cell viability, the larger will be the blue formazan deposits. This colored metabolite was dissolved in acid isopropanol (0.04 N HCl) and incubated for 1 h at room temperature. Absorbance was measured at 570 nm, subtracting the absorbance at 630 nm with a Bio-Rad iMarkTM Microplate reader (Bio-Rad Laboratories, Richmond, CA, USA). The results were expressed in absolute absorbance readings; a decrease in absorbance indicated a reduction in cell viability.

#### 3.3.4. Nitrite Determination

Nitric oxide (NO) levels were assessed by nitrite quantification, as described by Grisham *et al.* [60]. Briefly, murine RAW 264.7 macrophages were cultured with different dilutions of each extract in a 96-well microplate for 1 h, after which cells were stimulated with lipopolysaccharide (LPS) (Sigma-Aldrich Chemie). After 24 h of incubation, 100 μL of culture medium were mixed with Griess reagent (Sigma-Aldrich Chemie). The latter reacts with NO present in the medium to give a red color. Absorbance was read at 540 nm with a Bio-Rad iMark microplate spectrophotometer. Therefore, the absorbance values give an indication of the amount of NO present in the medium.

**Table 5:** Chlorogenic acid (CGA) content and ranking for CGA in the methanolic extracts of the eight accessions for which the biological activity (cytotoxicity and inhibition of NO production in RAW 264.7 macrophages) was evaluated.

Group	Code	CGA Content (g/kg)	CGA Rank
Scarlet eggplant			
Aculeatum	ACUL	1.02	6
*aethiopicum-anguivi*	AE-AN	1.40	5
Gilo	GILO1	0.21	8
Gilo	GILO2	3.69	2
Kumba	KUMB	0.23	7
Shum	SHUM	2.21	3
Gboma eggplant			
*S. dasyphyllum*	DASY	4.87	1
*S. macrocarpon*	MACR	1.98	4

### 3.4. Data Analysis

The mean, minimum and maximum values for the total reducing capacity, chlorogenic acid content and chlorogenic acid peak area of scarlet eggplant and for gboma eggplant accession means were calculated. Analysis of variance (ANOVA) tests were performed separately for accessions of scarlet eggplant and for accessions of gboma eggplant in order to calculate the sums of squares corresponding to accession and to residual, average standard error of accession means, coefficient of phenotypic variation (CV_P_; %), coefficient of genotypic variation (CV_G_; %) and broad-sense heritability (H^2^) (Table 1) [45]. Broad-sense heritability was calculated using the formula, 100 σ_G_^2^/σ_P_^2^, where σ_G_^2^ and σ_P_^2^ are, respectively, the genotypic and phenotypic variance calculated from ANOVA mean squares [45]. Mean values for each accession were used to perform additional ANOVA analyses to detect differences among group means (Table 2). Significant differences among accessions and among group means were detected using the Duncan multiple range test [50]. Pearson linear coefficients of correlation (*r*) and coefficients of determination (*r*^2^; %) between total reducing activity and chlorogenic acid content were calculated using accession means for all accessions, and their significance was studied with an F-test (Table 3) [50]. The total reducing capacity accounted for by chlorogenic acid (%) was calculated, and an ANOVA test was performed to detect differences among the different scarlet eggplant and gboma eggplant groups. The production of nitric oxide in the biological assays is expressed as the mean ± standard error values. Statistical significance was determined with an ANOVA followed by Dunnett’s *t*-test for multiple comparisons.

## 4. Conclusions

We have found that scarlet eggplant and gboma eggplants present a high diversity for reducing activity and for chlorogenic acid content, which is the main phenolic acid in these eggplants. Heritability values have been moderate to high for these traits, indicating that selection will be efficient. Considerable differences have been found among and within groups of scarlet and gboma eggplants for total reducing capacity, chlorogenic acid content and chlorogenic acid peak area, with *S. dasyphyllum* having the highest values for reducing capacity and chlorogenic acid content. In most of the groups, chlorogenic acid has been found to be correlated with reducing activity, indicating that it plays a main role in the bioactive properties of scarlet and gboma eggplants. The biological assays showed that gboma eggplants (in particular, *S. dasyphyllum*) extracts had a higher cytotoxicity than those of scarlet eggplants and that, in general, the higher the chlorogenic acid content, the higher the inhibition of NO production of LPS stimulated macrophage cells. The results obtained suggest that both crops have important bioactive properties and that selection and breeding in these crops can result in scarlet and gboma eggplants with enhanced reducing activity and chlorogenic acid content, as well as improved biological activity.
